# Experimental Investigation of Mechanical Behavior and Damage Evolution of Coal Materials Subjected to Cyclic Triaxial Loads with Increasing Amplitudes

**DOI:** 10.3390/ma18132940

**Published:** 2025-06-21

**Authors:** Zongwu Song, Chun’an Tang, Hongyuan Liu

**Affiliations:** 1 School of Resources and Civil Engineering, Northeastern University, Shenyang 110819, China; 2 State Key Laboratory of Coastal and Offshore Engineering, Dalian University of Technology, Dalian 116024, China; 3 School of Engineering, University of Tasmania, Hobart, TAS 7001, Australia; hong.liu@utas.edu.au

**Keywords:** coal behavior, cyclic loading, acoustic emission, damage evolution, failure pattern

## Abstract

As a part of the mining-induced stress redistribution process during coal mining, the repeated loading and unloading process with increasing peak stresses will cause more severe deformation and damage to mining roadways, which is different from the findings in other underground engineering practices. Consequently, cyclic triaxial compression tests with increasing amplitudes were carried out to investigate the mechanical behavior, acoustic emission (AE) characteristics, and damage evolution of coal materials. It is found that peak deviatoric stress and axial residual strain at the failure of coal specimens increase with increasing confining pressures, while the changes in circumferential strain are not obvious. Moreover, the failure patterns of coal specimens exhibit shear failure due to the constraint of confining pressures while some local tensile cracks occur near the shear bands at both ends of the specimens. After that, the damage evolution of coal specimens was analyzed against the regularity of AE counts and energies to develop a damage evolution model. It is concluded that the damage evolution model can not only quantify the deformation and failure process of the coal specimens under cyclic loads with increasing amplitudes but also takes into account both the initial damage due to natural defects and the induced damage by the cyclic loads in previous cycles.

## 1. Introduction

Coal or rock is a heterogeneous material containing many natural defects such as pores, microcracks, and joints. These internal defects evolve under external loads, causing damage accumulation associated with the initiation and propagation of microcracks in coal or rock materials. Thus, a macrocrack is not an instantaneous event that occurs at a certain level of stress but the culmination of a microcracking process [1]. Correspondingly, the cracking process at the micro-scale often has an important effect on the macroscopic performance of rock materials and is worthy of further investigation. The microcracking process of the rock materials is accompanied by acoustic emissions (AEs) that are defined as transient elastic waves generated by the sudden release of strain energy in this process. Therefore, since AEs are a natural by-product of microcrack growth and brittle fracturing, the analysis of AE signals is well suited to improve our understanding of the microcracking and failure processes of rock materials [2].

AE signals can be collected by the sensors attached to the surface of rock specimens or testing equipment, which provide a wealth of valuable information about rock damage and fracture, including fracture locations, source mechanisms, and stress levels involving the Kaiser effect [3,4,5]. The AE monitoring technique has been extensively used to quantitatively analyze the cracking process and cracking mode of rocks and concretes by AE parameters such as AE energy, AE events, waveform amplitude, AE average frequency, and the RA value (the ratio of the rise time to the waveform amplitude) [6,7,8,9]. In laboratory uniaxial compression tests, the variation in AE parameters during the loading process depends strictly on the damage to the rock specimens, and the energy content of AE signals increases when approaching the final failure of the specimens [10]. The three-dimensional location of AE events directly reflects the spatial evolutionary process of microcracks and the stress redistribution in the rock specimens under uniaxial compression [11]. Correspondingly, AE technique and theory have shown good applicability and non-destructive testing advantages not only in laboratory tests but also in underground excavation engineering field practices and numerical simulations for studying the fracture process and failure mechanisms of rocks and other brittle materials [12,13,14,15,16,17].

It is widely known that the mechanical behavior and failure characteristics of rocks depend strongly on external stress conditions. For example, the AE hit rate (the number of AE signals per second or per 1MPa stress increment) during uniaxial reloading of triaxially preloaded specimens begins to increase virtually from the beginning of the cyclic loading test, which is considerably different from the behavior observed after uniaxial preloading [3]. In practice, the stress redistribution caused by excavations in underground engineering can significantly affect the original properties of rock masses. For example, the repeated disturbance of complicated mining-induced stresses threatens the stability of mining roadways during coal mining. In a longwall mining process, the immediate roof strata are allowed to collapse behind the mining face [18]. After the sufficient collapse of the roof strata, the overlying strata above the caved zone fracture and move downward continually until the fractured strata and caved rocks are in contact to achieve new stress balances [19]. The periodic fracturing and movement of overlying strata with the advance of the mining face make the stress distribution in rock strata change continually and lead to repeated loading–unloading effects on the adjacent coal masses. Correspondingly, microcracks and macrocracks develop in the coal masses under cyclic loading, which cause the degradation of the physical and mechanical properties of the coal masses and the reduction in their bearing capacity. Some coal pillars designed to support the roadway and isolate the caved zone are affected by the superimposed mining-induced stresses from adjacent mining panels, and when this occurs, deformation and damage of the coal pillars are more severe.

The conventional cyclic loading tests usually adopt a constant amplitude waveform, such as the sinusoidal waveform, for loading at each cyclic stage [20,21,22,23]. However, during actual mining engineering practices, the maximum stress of each loading and unloading cycle on coal or rock masses is usually not strictly equal. It has been reported that the failure properties of rocks under cyclic loading depend on the fatigue threshold and amplitude, and rock failure may not occur no matter how many loading cycles are continued when the maximum stress is lower than a certain stress level [24]. However, further investigations are needed to clarify the effect of the fatigue threshold and amplitude of cyclic loads on the failure properties of rocks. Moreover, the disturbance stresses caused by coal mining sometimes have a superimposed effect, which means that the maximum stress of the next loading cycle may be higher than that of the previous one.

In this study, cyclic triaxial compression tests on coal specimens with increasing amplitudes were carried out to investigate the deformation and damage behavior of coal materials. The AE characteristics of the coal specimens were also studied to develop a damage evolution model. The findings aim to better reveal the failure mechanisms and characteristics of coal materials under mining-induced repeated loading and unloading and to provide support for the stability maintenance of mining roadways.

## 2. Experimental Procedure

### 2.1. Experimental Equipment and Specimens

**Figure 1:**
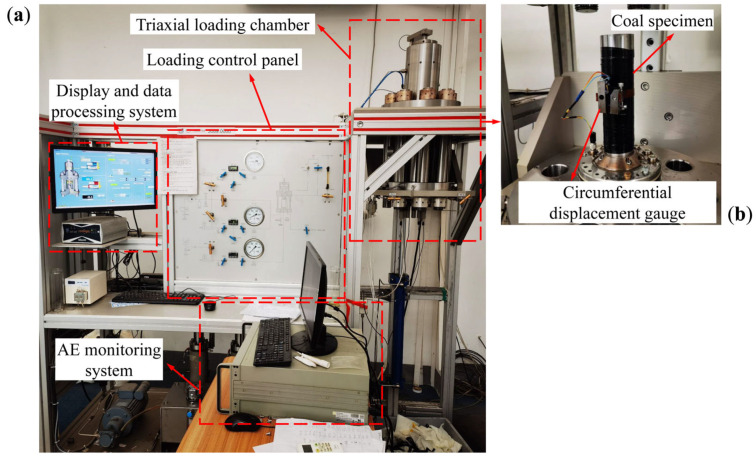
Experimental system: (**a**) STAC-600-600 multifunctional rock mechanics testing system; (**b**) coal specimen installation.

All the cyclic loading tests were carried out using the STAC-600-600 multifunctional rock mechanics testing system (developed by the corporation TOP INDUSTRIE, Vaux-le-Pénil, France), and the AE21C multichannel AE monitoring system was employed to collect and process the AE signals in real time during the tests, as shown in Figure 1. This testing system can provide a maximum axial load of 380 MPa, a maximum confining pressure of 60 MPa, and a maximum pore pressure of 40 MPa. Axial displacements were automatically recorded and processed in the system to measure axial deformations while circumferential deformations were measured by a reusable circumferential displacement gauge with a measuring capacity of 0–4 mm. Uniaxial and triaxial compression, cyclic loading and unloading, and rheological tests of rock materials under multi-field coupling conditions can be performed using this testing system. According to many debugging tests and the monitored background noise, the AE signals detected in the sensors were amplified by 35 dB and the threshold was set to 24 dB. The time parameters of the AE signals, including the Hit Definition Time and Hit Interval Time, were set to 50 and 300 μs, respectively. The AE signals were recorded at an acquisition rate of 1 MHz by 4-channel modules.

**Figure 2:**
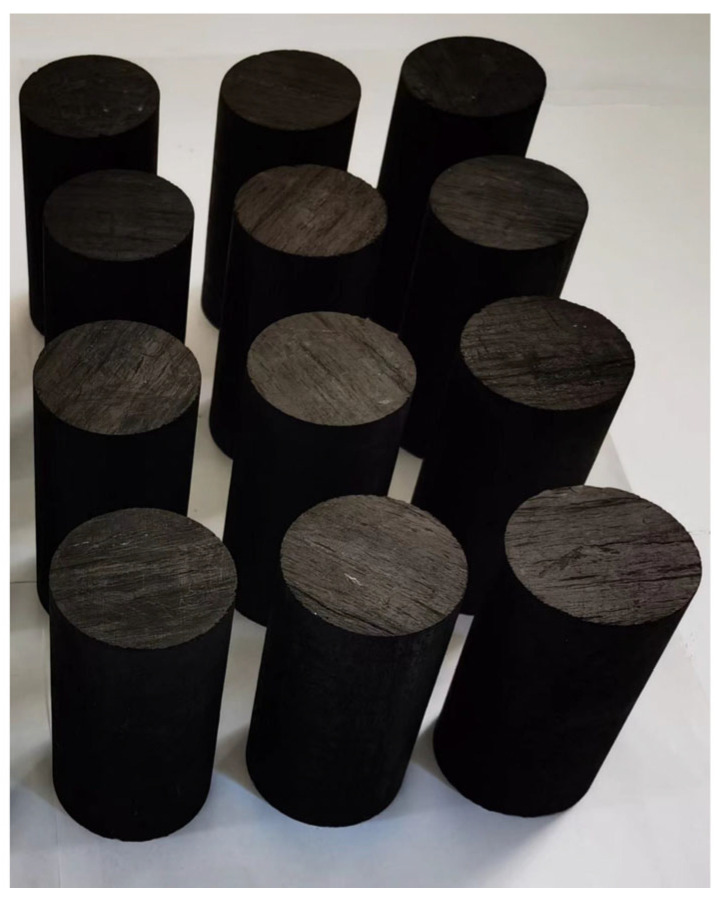
Partial coal specimens prepared for testing.

The coal specimens were taken from the Binchang coal mining field, Shaanxi Province, China, which were drilled from large coal blocks and cut into cylinders with a diameter of 50 mm and a length of 100 mm (Figure 2). In order to ensure the homogeneity of the coal specimens for the subsequent cyclic loading tests, non-destructive acoustic tests were conducted to select specimens with similar wave velocities. The uniaxial compressive strength of the coal specimens was in the range of 15–18 MPa after testing three without any visible macroscopic cracks, and the average compressive strength was 16.3 MPa. Poisson’s ratio of the coal specimen was 0.28 after measurement. The basic physical properties of the coal specimens are summarized in Table 1.

### 2.2. Experimental Method and Procedure

**Figure 3:**
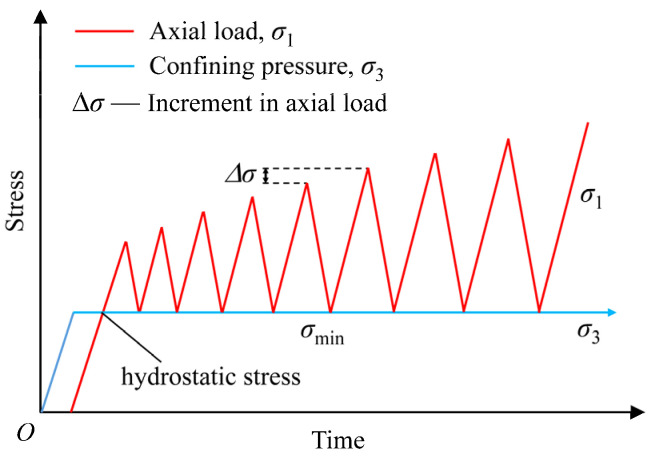
Loading path in cyclic triaxial compression tests.

To determine the cyclic loading method in these tests, we had to better understand the characteristics of stress redistributions caused by the periodic roof collapse during the active process of longwall coal mining and their influences on the surrounding rocks. The stress state of the roof and surrounding rocks during longwall mining can be reflected by the pressure changes in shield support against the roof [25]. The maximum value of the pressure in the support legs increases with the movement of the roof. The lower limit of the pressure in the support legs also increases to maintain a constant support height, which is different from the stress changes in the coal mass. Because the coal mass cannot provide self-adaptive support, the stress in the coal mass reverts to an initial level dependent on confinement conditions after unloading. Therefore, referring to the pressure changes in shield support against the roof and previous research on cyclic loading methods [26,27], a cyclic triaxial loading path with increasing amplitudes was carried out, as shown in Figure 3. Three different confining pressures of 6, 9, and 12 MPa were applied as examples in the cyclic triaxial loading tests. The triaxial compressive strength of the coal specimen was essential for the determination of different unloading levels in the cyclic loading tests. Three groups of conventional triaxial compression tests (three specimens in each group) were conducted under the preset confining pressures of 6, 9, and 12 MPa, and the average triaxial compressive strength obtained from these tests was 51.2, 56.3, and 60.7 MPa, respectively.

During the cyclic triaxial compressive tests, the confining pressure was first loaded at a constant rate on the coal specimen to the designated value by the stress control mode after the installation of the specimen, which remained constant throughout the test. The axial stress was loaded at a constant rate until 65% of the triaxial compressive strength of the coal specimen and then unloaded at the same rate to the corresponding confining pressure level. The first loading and unloading cycle was completed accordingly. After that, the peak stresses of the following cycles increased gradually in an equal load increment (Δ*σ*) until the specimen failed. To obtain enough cycles for the analysis of damage evolution in the pre-peak phase, the load increment between two adjacent cycles adopted 5% of the triaxial compressive strength. The minimum stress (*σ*_min_) of the unloading process in each cycle still maintained the corresponding confining pressure level. All the loading and unloading processes were completed linearly at the same rate of 0.1 MPa/s. The detailed cyclic loading path is shown in Figure 3.

## 3. Experimental Results and Analyses

### 3.1. Characteristics of Stress–Strain Curves

**Figure 4:**
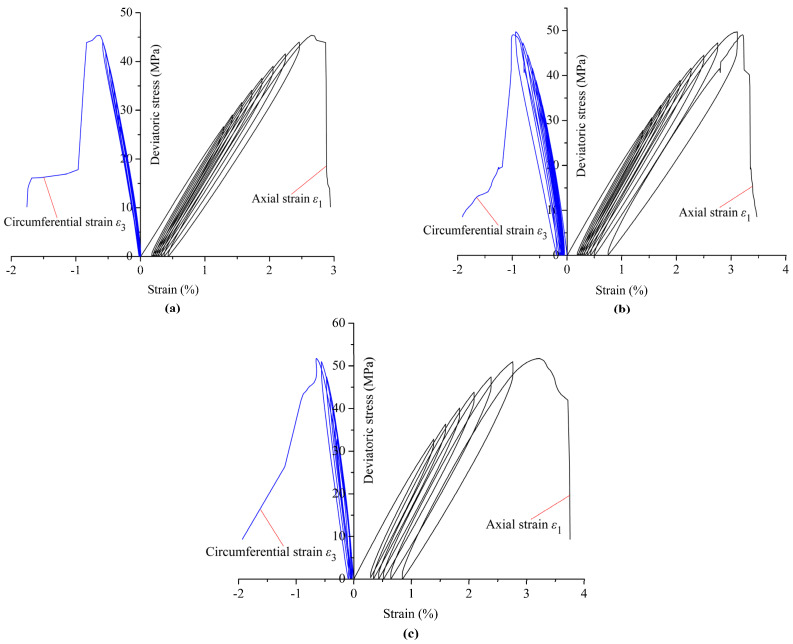
Deviatoric stress–strain curves of coal specimens under confining pressures of (**a**) 6 MPa, (**b**) 9MPa, and (**c**) 12 MPa.

A coal specimen with typical characteristics was selected from each group of the cyclic triaxial tests under the same confining pressure to analyze its deformation and failure behavior. These are the specimens C6-2, C9-4, and C12-7 under the three confining pressures of 6, 9, and 12 MPa, respectively. Figure 4 shows the deviatoric stress (*σ*_1_–*σ*_3_)–strain (*ε*) curves of the coal specimens under the three different confining pressures. It can be seen from Figure 4 that the peak deviatoric stress increases with increasing confining pressure, and, thus, the bearing capacity of the coal specimen is improved. The reason for this is that the high confining pressure can inhibit the initiation and expansion of cracks in the specimen [23]. The peak deviatoric stresses at the failure of the specimens under the three confining pressures of 6, 9, and 12 MPa are 45.38, 49.04, and 52.35 MPa, respectively, increasing by 15.36% from the lowest to highest confining pressures in the tests. The axial strain at failure also gradually increases with increasing confining pressure, reaching an increase of 21.43%. However, the circumferential strain at failure does not increase significantly with increasing confining pressure because the high confining pressure restricts the circumferential deformation of the coal specimens (Figure 4c). The deviatoric stress drops rapidly after reaching peak stress under low confining pressure (Figure 4a,b) but an increase in the axial strain is still maintained before the final stress drop under high confining pressure (Figure 4c). The transition from brittle to ductile deformation in coal or rock specimens may occur with increasing confining pressure if the confining pressure is high enough.

**Figure 5:**
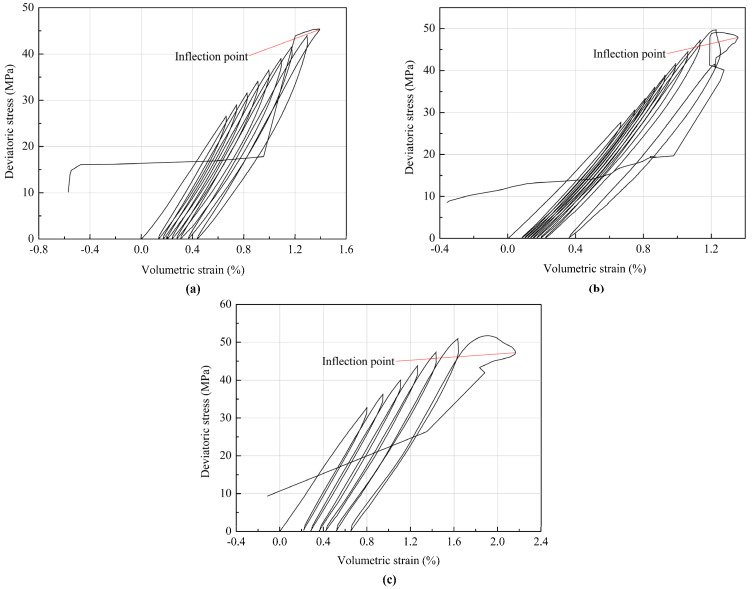
Deviatoric stress–volumetric strain curves of coal specimens under confining pressures of (**a**) 6 MPa, (**b**) 9MPa, and (**c**) 12 MPa.

The relationship between the deviatoric stress and volumetric strain of the coal specimens under different confining pressures is shown in Figure 5. The volumetric strain of the specimen under triaxial stress state is usually defined as Equation (1) [28]:

(1) $$\varepsilon_{v}=\varepsilon_{1}+2\varepsilon_{3}$$

where *ε*_v_, *ε*_1_, and *ε*_3_ are the volumetric, axial, and circumferential strains, respectively. The dilatancy boundary of rocks under compression can be defined as the turnover point or inflection point on the volumetric strain curve. The transition from crack closure and reduction in pore volume to crack reopening, microfracturing, and expansion of pore volume takes place near a dilatancy boundary [29]. The volumetric strain is controlled by both the axial strain and circumferential strain according to Equation (1). It can be observed from Figure 5 that the inflection points on the volumetric strain curves of all testing specimens are almost close to the peak deviatoric stresses at failure, which means that the circumferential deformation begins to dominate the volumetric deformation and then the dilatancy occurs when the coal specimen approaches failure. All coal specimens exhibit significant brittle failure characteristics, which is consistent with the argument that brittle rocks usually exhibit a lack of ability to sustain plastic deformation in the failure process [30]. Therefore, considering the observations in Figure 5, the confining pressure and brittleness of the coal specimen have a significant impact on the dilatancy behavior of the coal specimen. After the inflection point, cracks grow rapidly and coalesce into macroscopic fracture surfaces, and the specimen dilatancy accelerates as the load continues.

**Figure 6:**
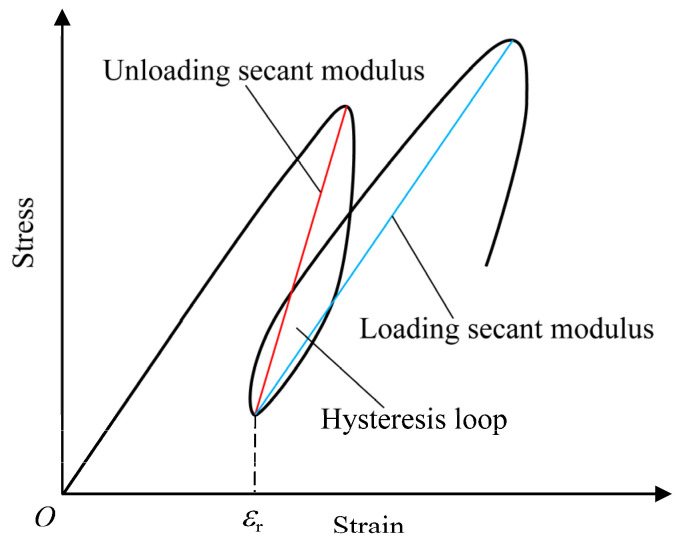
Definitions of hysteresis loop, secant modulus, and residual strain (*ε*_r_).

In the stress–strain curves of the coal specimens under cyclic loading and unloading, each unloading curve and the next loading curve together form a closed loop, known as a hysteresis loop, as shown in Figure 6. The formation of the hysteresis loop is due to the irreversible damage in the coal specimens during cyclic loading and unloading, such as the initiation and propagation of microcracks and friction between them, which also reflects the dissipation of energy in this process. The hysteresis loops shown in Figure 4 and Figure 5 change from dense to sparse and their area increases, indicating that the irreversible plastic deformation and damage accumulation increase with the continuous cyclic loading. The cyclic loading frequency and amplitude, and the discretization of the strength between coal specimens, might lead to an unequal number of hysteresis loops or cycles in the testing process.

### 3.2. Characteristics of Residual Strain and Secant Modulus

**Figure 7:**
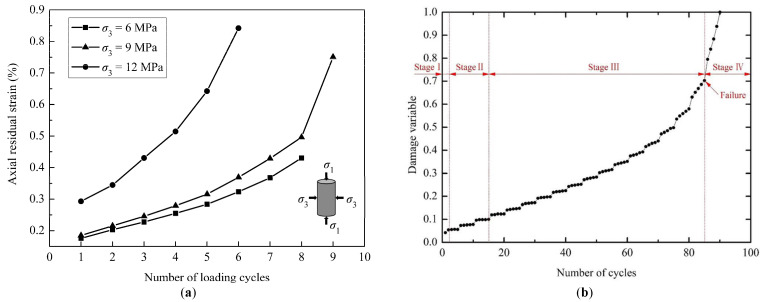
Comparison of experimental results: (**a**) variation in axial residual strain with the number of loading cycles; (**b**) variation in damage variable defined by the axial residual strain reported by Zhu et al. (2020) [23].

The residual strain (*ε*_r_) reflects the irreversible deformation of the coal specimens after unloading, which is defined as the strain at the end of each unloading cycle (Figure 6). The variations in the axial residual strain with the number of loading cycles are given in Figure 7a. At the initial stage of the loading cycles, the axial residual strain increases slowly, especially for the specimens under relatively low confining pressures. As the number of loading cycles increases, the axial residual strain increases rapidly, and a sharp increase in the axial residual strain occurs in the ultimate failure process of the specimens. Therefore, the increasing rate of the axial residual strain becomes higher as the cyclic load continues. The specimen failure under cyclic loading is a result of progressive accumulation of damage to failure. The law obtained from Figure 7a is almost consistent with the variation in the damage variable defined by the axial residual strain reported by Zhu et al. (2020) (Figure 7b) [23]. It can also be observed from Figure 4 and Figure 7 that the axial residual strain at the end of the final unloading cycle increases with increasing confining pressure but the increase is not obvious for the circumferential residual strain.

**Figure 8:**
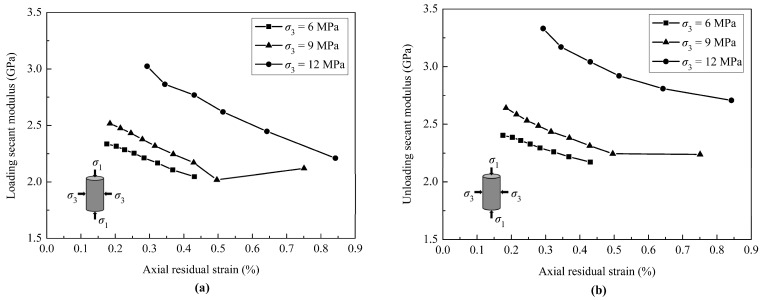
The curves of secant modulus versus axial residual strain under different confining pressures: (**a**) loading stages; (**b**) unloading stages.

The secant modulus (*E*_sec_) is defined as the slope of the line connecting the highest and lowest points of each cycle, including the loading secant modulus and unloading secant modulus, as shown in Figure 6. The secant modulus is one of the representative parameters for describing the deformation characteristics of rocks. The relationship between the secant modulus and the axial residual strain under different confining pressures is given in Figure 8, which shows that the loading secant modulus decreases as the axial residual strain increases, regardless of the confining pressure. This indicates that the deformation resistance of the coal specimen is gradually weakened as the cyclic load continues. The variation trend of the unloading secant modulus with the axial residual strain is similar to that of the loading secant modulus. However, under the same axial residual strain, the unloading secant modulus is greater than the loading secant modulus.

### 3.3. Failure Patterns of Coal Specimens

**Figure 9:**
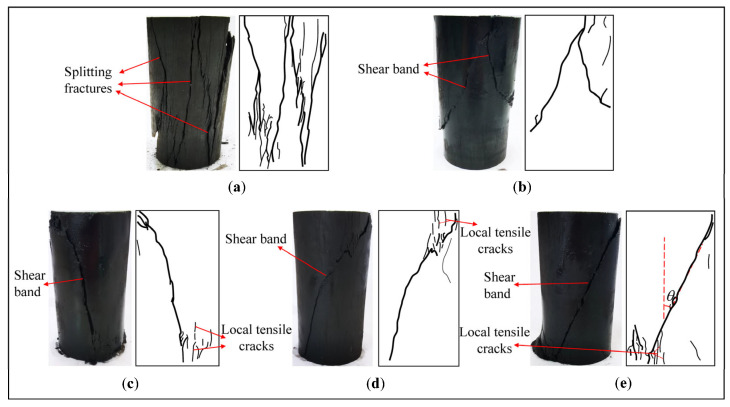
Failure patterns of coal specimens after various tests: (**a**) uniaxial compression; (**b**) conventional triaxial compression; (**c**–**e**) cyclic triaxial compressions with increasing amplitudes—the confining pressure is 6, 9, and 12 MPa, respectively.

Figure 9 shows the failure patterns of the coal specimens after the cyclic loading tests. The trace depictions of fracture bands and cracks on the specimen surface are also presented to illustrate the failure patterns clearly. Under uniaxial compression, the axial splitting fractures and some inclined cracks on the specimen surface can be observed in Figure 9a. The specimen failure under uniaxial compression is mainly induced by extensile fractures almost parallel to the applied axial stress or major principal stress [31]. Under triaxial stress conditions, the failure patterns of the specimens are mainly manifested by one or more shear fracture planes or bands (Figure 9b–e). The reasons are that the high confining pressure inhibits the expansion of individual microcracks and leads to the coalescence of a large number of microcracks into a shear fracture zone at higher loads [32]. However, compared with the conventional triaxial compression (Figure 9b), more local tensile cracks occur near the shear bands on the upper and lower ends of the specimens under the cyclic triaxial compressions (Figure 9c–e). The tensile cracks occurring in the coal specimens can also be found in the cyclic triaxial loading tests by Zhang et al. [26]. The microcracks in the specimen will open, close, and develop continuously under cyclic loading, and the initiation and propagation of these tensile cracks are driven by a portion of the input energy from the loading system. In addition, the angle (*θ*) between the shear band and axial direction or major principal stress changes with the confining pressure changing (Figure 9b,c), which can be used to describe the shear failure characteristics of the coal specimen. The orientation of the shear bands with respect to the axial direction increases with increasing confining pressure [33]. Sometimes it is difficult to measure the shear fracture angle (*θ*) accurately in laboratory tests because of the irregular geometry of the macroscopic fracture plane induced by the heterogeneity of coal materials. Moreover, the failure patterns of the specimens are sensitive to local variations in the mechanical properties of the specimens (local heterogeneity) [32].

### 3.4. AE Characteristics and Damage Evolution of Coal Specimens

#### 3.4.1. Analysis of AE Characteristics

The generation of AE signals is generally related to the initiation and propagation of internal microcracks in brittle materials such as coal and rock. The microcracking process of the materials can be analyzed from a wealth of information contained in the AE signals. In this study, two typical AE parameters, i.e., AE counts and AE energy, are used to reveal the damage and failure processes of the coal specimens under cyclic triaxial loading. The AE counts and AE energy can be used to describe the frequency and relative intensity, respectively, of the microseismic activities associated with the deformation and damage of the specimen.

**Figure 10:**
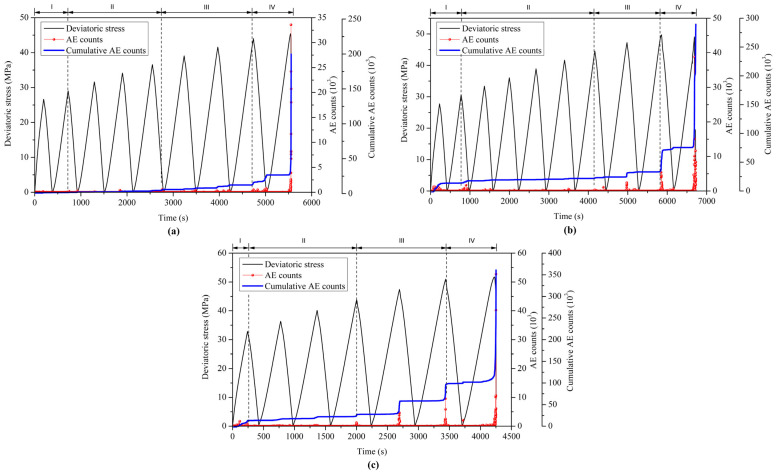
Characteristics of AE counts in cyclic triaxial compression tests under confining pressures of (**a**) 6 MPa, (**b**) 9 MPa, and (**c**) 12 MPa.

**Figure 11:**
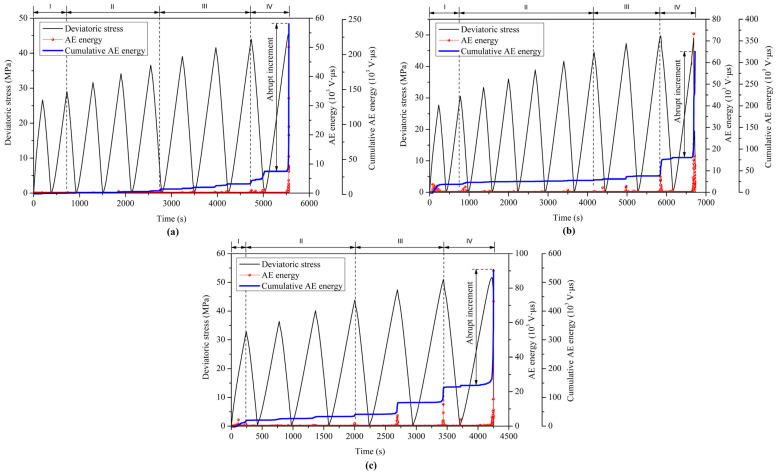
Characteristics of AE energy in cyclic triaxial compression tests under confining pressures of (**a**) 6 MPa, (**b**) 9 MPa, and (**c**) 12 MPa.

Figure 10 and Figure 11 show the AE characteristics of coal specimens during the cyclic loading tests under three different confining pressures. The AE parameters exhibit different characteristics at various stages corresponding to the deformation and failure processes of the coal specimens. The multi-stage descriptions of the AE parameters of rocks have also been reported in previous experimental studies [23,26,34] but differ from those of this study due to the different experimental methods and specimen materials. The AE responses of the coal specimens under cyclic triaxial compression with increasing stress amplitudes are unclear and need further studies. As presented in Figure 10, in the initial stage of cyclic loading (I), the original pores and microcracks in the coal specimens are compacted and closed, resulting in a small number of AE counts. This initial compaction process usually lasts for a short period of time. The degree of AE activity in the initial compaction stage depends strongly on the original defect density and damage state of the coal specimens. As the cyclic axial load continues, the elastic behavior begins to dominate the stress–strain relationship after the compaction stage. The second stage of cyclic loading (II) usually corresponds to the quasi-elastic deformation stage in the stress–strain relationship of the coal specimen. There is almost no significant increase in AE counts at this stage because the load level is not sufficient to cause the initiation of new microcracks. The curves of cumulative AE counts show approximately horizontal linearity in the quasi-elastic deformation stage, indicating that the AE activity in the coal specimens remains relatively quiet. However, the crack closure or opening can take place without disturbing the linearity in the stress–strain behavior [29]. After stage II, the irreversible plastic deformation of the coal specimens increases with the continuous cyclic axial loading. The initiation and growth of numerous microcracks occur in the third stage of cyclic loading (III), resulting in a relatively large number of AE counts. The AE activity begins to be more active in stage III, and the demonstrable Kaiser effect [35] can be found in this stage, as shown in Figure 10b,c. The frictional motion between the flanks of existing cracks may generate repeated AE signals by the stick and slip processes during the repeated loading of a specimen [36]. In the final stage of cyclic loading (IV), these microcracks inside the coal specimens gradually coalesce and propagate to form macroscopic fractures, ultimately causing specimen failure. The AE counts and cumulative AE counts increase rapidly in the final stage due to the development of microcracks and macroscopic fractures and reach their maximum values near the peak stress at failure. It can be seen from Figure 10 that the peak values of AE counts and cumulative AE counts gradually increase with increasing confining pressure. The evolution of AE counts corresponds to the complete failure process of the coal specimen from damage accumulation to macroscopic fracture.

The characteristics of AE energy are similar to those of AE counts during cyclic loading tests, as shown in Figure 11. The AE energy is usually proportional to the squared waveform amplitude of an AE event or signal [37,38], reflecting the relative intensity of the released energy during the fracturing process. The AE energy is sometimes defined as the area under the envelope curve of voltage versus time [9,39]. In the initial compaction and quasi-elastic stages (I and II), the AE energy is at a low level and the curves of the cumulative AE energy remain flat at a low level. However, the AE energy and cumulative AE energy begin to increase rapidly in the stages of microcrack growth and macroscopic fracture (III and IV), and they increase abruptly to the maximum level when the coal specimens fail. The abrupt increment in AE energy in the final loading cycle can be determined by the difference between the maximum value and the last flat level of the cumulative AE energy (Figure 11). These abrupt increments in AE energy under three confining pressures of 6, 9, and 12 MPa account for 86.47%, 74.53%, and 73.28% of the total cumulative AE energy, respectively. Therefore, most of the released AE energy occurs in the macroscopic fracture process induced by rapid crack development in the final loading cycle, although the previous accumulation of irreversible damage like microcrack growth contributes to the occurrence of this process. The released AE energy in the damage and failure processes of the coal specimens increases with increasing confining pressure. Under high confining pressure, more input energy is required to drive the specimen to fail and, correspondingly, the release of more strain energy stored inside the specimen leads to more severe failure of the specimen.

#### 3.4.2. Damage Evolution

Since the AE signals are the transient elastic waves accompanying the localized microcracking in a material during the deformation process, they will inevitably be associated with the evolution of defects within the material. The AE count can reflect the microcracking features and damage extent within materials such as coal [40], and it agrees with the damage parameters used for describing the evolution of the intra-material defects. Accordingly, we will deal with a quantitative analysis of damage evolution in the coal specimen depending on the regularity of AE counts during cyclic loading tests.

Considering the effects of the damaged area or microdefects on the deterioration of the mechanical properties of the coal specimen, the damage variable can be defined as follows [41,42]:

(2) $$D=\frac{A_{d}}{A}$$

where *D* is the damage variable; *A_d_* is the damaged area on the cross-section of a coal specimen; and *A* is the cross-sectional area of the initial undamaged specimen. The changes in *D* from 0 to 1 represent a state transition from no damage to complete failure of the specimen. The AE count rate per unit area of the damaged section can be defined as follows:

(3) $$c=\frac{C_{m}}{A}$$

where *c* is the AE count rate; and *C_m_* is regarded as the total AE counts when the overall cross-sectional area *A* is completely damaged. Thus, the cumulative AE counts (*C*) when the damaged area of the specimen reaches *A_d_* can be expressed as outlined below:

(4) $$C=c\times A_{d}=\frac{C_{m}}{A}\times A_{d}$$

Combining Equations (2) and (4), the damage variable can be expressed by the cumulative AE counts as follows:

(5) $$D=\frac{C}{C_{m}}$$

Equation (5) indicates that the cumulative AE counts agree with the damage variable and can quantitatively describe the degree of damage accumulation in the specimen. It should be noted that the damage variable represented by the cumulative AE counts depends on the voltage threshold of the AE acquisition. Considering the Kaiser effect of AE in the repeated loading experiments, the damage variable *D_i_* for the *i*-th cycle can be described by Equation (6):

(6) $$D_{i}=\frac{C_{i}}{C_{m}}$$

where *C_i_* denotes the cumulative AE counts in the *i*-th cycle. When the stress level in the *i*-th cycle is lower than the damage stress threshold in the previous cycles—and thus insufficient to generate new AE counts—the damage variable *D_i_* is equal to 0.

Krajcinovic and Silva [43] proposed that the actual damage law in brittle specimens conforms to the Weibull distribution of the rupture strengths of individual elements in the specimens. The regularity of AE activities associated with the damage evolution is in essence a statistical one. Moreover, the damage accumulation is always related to irreversible strain at the microscopic level [44]. Thus, a relationship between the cumulative AE counts in the *i*-th cycle and strain distribution is given by the following:

(7) $$\frac{C_{i}}{C_{m}}=H_{i}\int_{\xi_{i-1}}^{\varepsilon }f(x)d(x)$$

where *ε* is the axial strain; *ξ_i_*_−1_ is the maximum strain that causes damage in the *i*−1-th loading cycle; and *f*(*x*) is a probability density function; in addition, *H_i_* is a unit step function, which can be defined as follows [44]:

(8) $$H_{i}=H(\varepsilon -\xi_{i-1})=\left\{\begin{array}{c} 1,\varepsilon \geq \xi_{i-1}\\ 0,\varepsilon <\xi_{i-1} \end{array}\right.$$

Many researchers [40,45,46,47] adopted the Weibull distribution to describe the distributional characteristics of micro-defects within materials, and its probability density function is outlined below:

(9) $$f(\alpha )=\frac{m}{\alpha_{0}}{\left(\frac{\alpha }{\alpha_{0}}\right)}^{m-1}\exp\left[-{\left(\frac{\alpha }{\alpha_{0}}\right)}^{m}\right]$$

where *α* is the material parameter of the element (such as strength, strain, or elastic modulus); the scale parameter *α*_0_ is related to the average value of the corresponding parameter; and *m* is the shape parameter of the Weibull distribution. If each element in the specimen is considered elastic, Equation (9) can be written as a strain distribution *f*(*ε*). Therefore, combining Equations (6)–(9), the damage variable for the *i*-th cycle can be written as follows:

(10) $$D_{i}=\frac{C_{i}}{C_{m}}=H_{i}\left\{\exp\left[-{\left(\frac{\xi_{i-1}}{\varepsilon_{0}}\right)}^{m}\right]-\exp\left[-{\left(\frac{\varepsilon }{\varepsilon_{0}}\right)}^{m}\right]\right\}$$

where *ε*_0_ is the average strain corresponding to the peak stress. Equation (10) is also a function of the Kaiser effect. The cumulative damage associated with the number of loading cycles can be obtained by accumulating all the damage within certain loading cycles using Equation (10).

**Figure 12:**
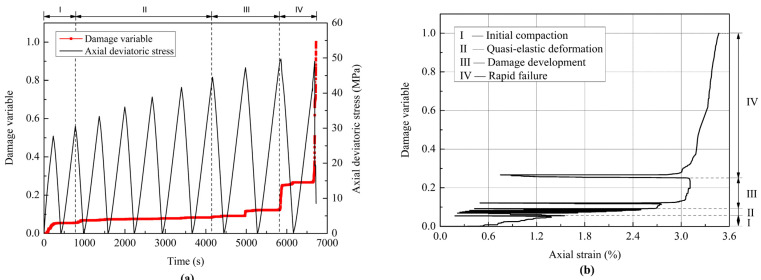
Damage evolution of the coal specimen subjected to cyclic loading under the confining pressure of 9 MPa: (**a**) damage variable versus testing time; (**b**) damage variable versus axial strain.

Here, we take one of the confining pressure conditions as an example to analyze the damage evolution in the coal specimen under cyclic loading, as shown in Figure 12. The changes in the damage variable are consistent with those of the cumulative AE counts in Figure 10. The damage variable increases slightly in the initial stage of cyclic loading (I), which is attributed to the closure of some primary pores and microcracks and the friction between them with increasing compaction. As the loading cycle increases, the damage variable remains almost unchanged during the period of 1000–4500 s, as shown in Figure 12a. This phenomenon is due to the fact that the coal specimen maintains the quasi-elastic deformation in the second stage (II) and there is little AE activity in the specimen. Subsequently, the damage variable begins to increase rapidly in the third stage (III) with the growth and propagation of microcracks in the specimen, and it increases significantly at the maximum stress in the last cycle of the third stage. In the final stage (IV), the damage variable increases sharply with the occurrence of macroscopic fractures and the eventual failure of the specimen. Correspondingly, the relationship between the damage variable and axial strain from the testing data shows similar multi-stage characteristics, including initial compaction, quasi-elastic deformation, damage development, and rapid failure, as shown in Figure 12b. When the axial strain decreases, the damage variable remains nearly constant, manifesting as approximately horizontal lines in Figure 12b. When the axial strain increases and exceeds the strain threshold that induces damage, the damage variable begins to increase, particularly in stages III and IV. Actually, it can be observed from Figure 12 that the coal specimen undergoes a progressive damage accumulation before its final sudden failure. It is the damage accumulation that brings about the failure of the heterogeneous coal specimens under cyclic loading.

## 4. Discussion

**Figure 13:**
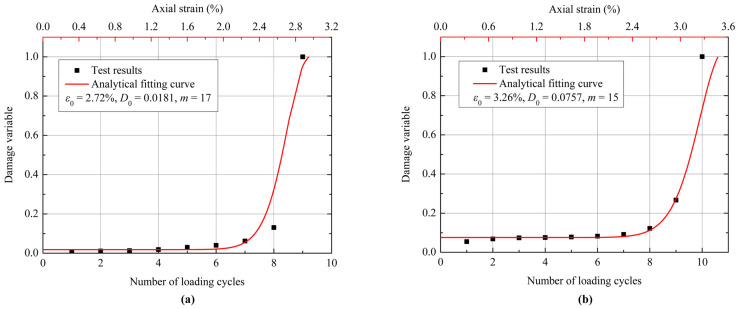
Relationship between the damage variable and the number of loading cycles, under confining pressures of (**a**) 6 MPa and (**b**) 9 MPa, and their fitting results.

The macroscopic fracture of the coal specimen can be regarded as the synthetic manifestation of the evolution of many microscopic cracks, which is an irreversible process of damage accumulation and can be described using a damage variable. Moreover, an important aspect of rock deformation and failure is the statistical distribution of weaknesses in the initial microstructure. Based on the Weibull distribution mentioned above, the cumulative distribution function reflecting the damage law (*D*(*ε*)) under the conventional loading conditions is given by the following [40]:

(11) $$D(\varepsilon )=F(\varepsilon )=\int_{0}^{\varepsilon }f(x)d(x)=1-\exp\left[-{\left(\frac{\varepsilon }{\varepsilon_{0}}\right)}^{m}\right]$$

where *f*(*x*) is the probability density function expressed by Equation (9); and the other parameters are the same as those explained above. It is clear that the damage variable *D*(*ε*) is equal to 0 when the axial strain *ε* is equal to 0. That is to say, there is no damage in the coal specimen before the deformation occurs. However, the original coal specimen is a heterogeneous material that contains many natural defects at different scales. Hence, a modified damage evolution model taking the initial degree of damage into account is proposed as Equation (12):

(12) $$\sum_{1}^{i}\frac{C_{i}}{C_{m}}=D(\varepsilon )=D_{0}+1-\exp\left[-{\left(\frac{\varepsilon }{\varepsilon_{0}}\right)}^{m}\right]$$

where *D*_0_ is related to the initial degree of damage of the coal specimen; and *m* is a fitting coefficient that defines the shape of the model curve. According to the experimental and fitting results shown in Figure 13, the values of *ε*_0_, *D*_0_, and *m* are 2.72%, 0.0181, and 17, respectively, when the confining pressure is 6 MPa. When the confining pressure is 9 MPa, the values of *ε*_0_, *D*_0_, and *m* are 3.26%, 0.0757, and 15, respectively. The value of *D*_0_ can be approximately determined as the average degree of damage after the compaction of the specimen. It should be noted that the damage evolution model in Equation (12) is only a rough mathematical description of the macroscopic damage process of materials and cannot describe the residual deformation and strength of materials after failure. The degradation treatment on the properties of the damaged materials may be required to describe the comprehensive mechanical behavior of the materials.

According to the definition of the damage variable in Equation (10), the damage accumulation in each cycle during the cyclic loading process can also be calculated. The loading process in each cycle can be regarded as a monotonic loading on the specimen with initial damage. Figure 13 shows the damage evolution of the coal specimens with increasing loading cycles from the experiments and their fitting curves obtained by Equation (12). It can be seen from Figure 13 that the cumulative damage law obtained by the proposed damage model is in good agreement with that from the experiments under cyclic loading conditions. An obvious feature in Figure 13 is that there is a sharp increase in the damage variable before the specimen failure, indicating that the microcracks propagate and coalesce rapidly within the specimen to form macroscopic fractures in the ultimate failure process.

It should be pointed out that the proposed damage evolution model is discussed here in light of the experimental results of the coal specimens, which should be applied to more types of rock materials in future studies. Moreover, it should be noted that the discussion in this paper focuses on the pre-peak failure behaviors of the coal specimens subjected to cyclic loading with increasing amplitudes. In addition, the stress-controlled loading mode is adopted in the cyclic loading experiments. Thus, after the peak stress, the deviatoric stress–strain curve will present a sharp stress drop in the post-peak phase under the stress-controlled loading mode.

## 5. Conclusions

In this paper, the mechanical behavior, AE characteristics, and damage evolution of coal specimens were investigated by cyclic triaxial loading tests with increasing amplitudes and AE monitoring. Compared with the conventional cyclic loading tests with a constant amplitude, the cyclic triaxial loading tests with increasing amplitudes can better simulate the repeated loading and unloading process of coal masses caused by longwall coal mining in field practice. On the basis of the evolution of the monitored AE count and energy, a damage evolution model is proposed to quantify the damage and failure process of the coal specimens under the cyclic triaxial loading tests with increasing amplitudes. The proposed damage evolution model can consider not only the initial damage state due to natural defects within the coal specimens but also that induced by the cyclic loads in previous cycles. The following conclusions can be drawn from this study:

The circumferential deformation begins to dominate the volumetric deformation and then the dilatancy occurs when the coal specimen in the cyclic triaxial tests approaches failure. The coal specimens exhibit significant brittle failure characteristics. The high confining pressure inhibits the initiation and propagation of microcracks in coal specimens, which eventually improves the bearing capacity of coal specimens. Moreover, the hysteresis loops change from dense to sparse and their area increases in the deviatoric stress–strain curves, reflecting that the irreversible deformation and damage in the coal specimens increase with the continuous cyclic loading.

The increasing rate of axial residual strain becomes higher with the increasing number of loading cycles. As the number of loading cycles increases, the secant modulus decreases with increasing axial residual strain, and the deformation resistance of the coal specimen is gradually weakened. In addition, the failure patterns of coal specimens exhibit shear failure accompanied by local tensile cracks near the shear bands at both ends of the specimens. The angle between the shear band and the axial direction increases with increasing confining pressure.

The evolution of the monitored AE count and AE energy corresponds to the complete deformation and failure processes of the coal specimens under cyclic loading. Most of the released AE energy occurs in the macroscopic fracture process during the final loading cycle but the previous accumulation of microcracks contributes to the occurrence of this process. The cumulative AE counts and released AE energy gradually increase with increasing confining pressure in the deformation and failure processes of the coal specimens under cyclic loading with increasing amplitudes.

The damage evolution of the coal specimens was quantitatively analyzed based on the monitored cumulative AE counts, which can be divided into four stages: initial compaction, quasi-elastic deformation, damage development, and rapid failure. The statistical description of the damage evolution in the coal specimen was discussed in light of the proposed damage evolution model, which was found to agree well with the experimental results. It is the damage accumulation that brings about the failure of the heterogeneous coal specimens under cyclic loading.

## Figures and Tables

**Table 1:** Physical properties of coal specimens and confining pressure conditions.

Specimen No.	Diameter/mm	Length/mm	*P*-Wave Velocity/m·s^−1^	*S*-Wave Velocity/m·s^−1^	Density/kg·m^−3^	Confining Pressure/MPa
C6-1	50.08	100.06	1986.92	1012.64	1298.70	6
C6-2	50.12	99.94	1968.43	1102.82	1278.33	6
C6-3	49.92	100.12	2008.25	1068.23	1247.77	6
C9-4	50.34	100.26	1993.23	1055.45	1265.52	9
C9-5	50.26	100.08	1988.56	1106.23	1282.34	9
C9-6	50.18	100.14	1982.43	1085.36	1275.66	9
C12-7	49.84	100.32	2000.65	1092.41	1268.14	12
C12-8	50.16	99.86	2002.43	1103.88	1273.43	12
C12-9	50.22	100.06	1995.82	1076.47	1275.35	12

## Data Availability

The original contributions presented in this study are included in the article. Further inquiries can be directed to the corresponding authors.
